# Genome-Wide Identification of Sweet Orange WRKY Transcription Factors and Analysis of Their Expression in Response to Infection by *Penicillium digitatum*

**DOI:** 10.3390/cimb45020082

**Published:** 2023-02-03

**Authors:** Dengxian Xi, Tuo Yin, Peichen Han, Xiuyao Yang, Mengjie Zhang, Chaojin Du, Hanyao Zhang, Xiaozhen Liu

**Affiliations:** 1 Key Laboratory for Forest Resources Conservation and Utilization in the Southwest Mountains of China, Ministry of Education, Southwest Forestry University, Kunming 650224, China; 2 Key Laboratory of Biodiversity Conservation in Southwest China, National Forest and Grassland Administration, Southwest Forestry University, Kunming 650224, China

**Keywords:** sweet orange, WRKY transcription factor, biological stress, *Penicillium digitatum*, genome-wide analysis

## Abstract

WRKY transcription factors (TFs) play a vital role in plant stress signal transduction and regulate the expression of various stress resistance genes. Sweet orange (*Citrus sinensis*) accounts for a large proportion of the world’s citrus industry, which has high economic value, while *Penicillium digitatum* is a prime pathogenic causing postharvest rot of oranges. There are few reports on how CsWRKY TFs play their regulatory roles after *P. digitatum* infects the fruit. In this study, we performed genome-wide identification, classification, phylogenetic and conserved domain analysis of CsWRKY TFs, visualized the structure and chromosomal localization of the encoded genes, explored the expression pattern of each *CsWRKY* gene under *P. digitatum* stress by transcriptome data, and made the functional prediction of the related genes. This study provided insight into the characteristics of 47 CsWRKY TFs, which were divided into three subfamilies and eight subgroups. TFs coding genes were unevenly distributed on nine chromosomes. The visualized results of the intron-exon structure and domain are closely related to phylogeny, and widely distributed cis-regulatory elements on each gene played a global regulatory role in gene expression. The expansion of the CSWRKY TFs family was probably facilitated by twenty-one pairs of duplicated genes, and the results of Ka/Ks calculations indicated that this gene family was primarily subjected to purifying selection during evolution. Our transcriptome data showed that 95.7% of *WRKY* genes were involved in the transcriptional regulation of sweet orange in response to *P. digitatum* infection. We obtained 15 differentially expressed genes and used the reported function of *AtWRKY* genes as references. They may be involved in defense against *P. digitatum* and other pathogens, closely related to the stress responses during plant growth and development. Two interesting genes, *CsWRKY2* and *CsWRKY14*, were expressed more than 60 times and could be used as excellent candidate genes in sweet orange genetic improvement. This study offers a theoretical basis for the response of CSWRKY TFs to *P. digitatum* infection and provides a vital reference for molecular breeding.

## 1. Introduction

In the world citrus production, the total yield of oranges can reach 75.459 million tons with a harvested area of 388.5 ha, with sweet orange (*Citrus sinensis*) accounting for the largest share that is the main orange juice processing variety with a high economic value (Food and Agriculture Organization of the United Nations, 2020). Recently, increased global warming has raised the risk of pathogen infection in orchards, and the various biotic and abiotic stresses cause high loss rates in sweet oranges [1], with *Penicillium digitatum* being the prime source of postharvest rot fruits [2]. In response to various environmental challenges, transcription factors (TFs) regulate plants to adapt to adversity stresses. TFs are specific proteins with at least two different domains in their structure, which can be divided into universal and specific transcription factors, which can interact with regulatory elements in the corresponding gene promoter regions, activating or inhibiting the expression of target genes [3,4].

WRKY, ERF, and bZIP were regarded as the prime disease-resistance families [5]. In recent years, numbers of WRKY TFs of non-model plant species have been identified in detail based on public databases and bioinformatic analysis. For example, in dicotyledonous plants, 54 AtruWRKY [6], 108 HbWRKY [7], and 56 CsWRKY [8] TFs have been identified in *Acer truncatum* Bunge, *Hevea brasiliensis*, and *Camellia sinensis*; among the monocotyledon plants, 164 MaWRKY [9], 86 BdWRKY [10], and 54 AcWRKY [11] TFs have been identified in *Musa nana* Lour., *Brachypodium distachyon*, and *Ananas comosus*. The WRKY families are identified and classified according to the number of heptad domains (WRKYGQK) and the characteristics of the zinc finger domain [12]. They are generally composed of 60 amino acids, and the WRKY domain is composed of heptad domains at the N-terminal, C2H2-type (C-X_4-5_C-X_22-23_-H-X-H, with X being an arbitrary amino acid) or C2HC-type (C-X_7_-C-X_23_-H-X-C) zinc finger domain can exist in the C-terminal or N-terminal. They are composed of about 30 amino acids and are a WRKY TFs DNA-binding domain functional region that can specifically recognize and bind to cis-regulatory elements [13,14]. In plants, the heptad domains often have one–two mismatched amino acids, which have also been identified as a member of the WRKY families [15].

In terms of classification, there are mainly two ways: Thomas Eulgem et al. [16] first classified the WRKY families into three classes based on the number of heptad domains and zinc finger structural features in the absence of a complete WRKY TF family in extant plants: Group I (2 WRKY domains, C2H2-type), Group II (1 WRKY domain, C2H2-type), and Group III (1 WRKY domain, C2HC-type). Rushton et al. [17] analyzed the intact domains in four model plants (*Glycine max*, *Oryza* sativa, *Arabidopsis thaliana,* and *Populus trichocarpa*), combined them with phylogenetic tree finding WRKY TF families more accurately classified as Group I, Group IIa + IIb, Group IIc, Group IId + IIe, and Group III. However, in numerous studies, the former classification method is still dominant, but the second classification method can more accurately reflect the evolutionary clustering relationship.

WRKY TFs regulate various signaling cascades that mediate different physiological responses in plants [18]. The binding of various environmentally induced factors to receptors on the cell membrane triggers the activation of signaling cascade systems such as MAPK, which phosphorylates WRKY proteins, enhances binding to W-box, regulates the expression of relevant functional genes, then participates in various vital physiological responses in plants [19]. In green tissues, signals such as TFs, light signals, and hormones form a complex network that maintains a costly balance between plant growth and defense that plays a vital role in regulating TFs activity and translocation in plant defense [20]. Stressed by adversity, the hormones in the body (e.g., Jasmonic acid JA, Salicylic acid SA, Auxin, Abscisic acid ABA and Ethylene ET) are altered, triggering a series of physiological activities that produce adaptations to resist stresses [21,22]. Numerous previous studies have shown the WRKY TFs are vital for responding to abiotic stresses (e.g., salt stress, drought stress, low-temperature stress, and heavy metal stress), biotic stress (e.g., *Pseudomonas syringae*, *Botrytis cinerea*, and virus) and in regulating growth and development (e.g., seed germination, leaf senescence). For example, the high expression of the *ZmWRKY106* gene in *Arabidopsis* can effectively improve the drought and heat tolerance of plants [23]; *WRKY4* and *WRKY12* genes of lily induced to be overexpressed under salicylic acid and Me-JA stressing that makes plants resist *B. cinerea* [24]; wheat WRKY42-B positively regulates the Me-JA induced leaf senescence process [25].

The growth and development of sweet orange and its stress response is a very complex biological process that depends on numerous gene interactions, as well the transcriptional regulations of WRKY TFs in response to stresses and plant signaling pathways play indispensable roles. However, there are few reports on the roles of CSWRKY TFs in the infection of fruits by *P. digitatum*. In this study, we performed genome-wide identification, classification, phylogenetic and conserved domain analysis of CsWRKY TFs, visualized the structure and chromosomal localization of the encoded genes, explored the expression pattern of each *CsWRKY* gene under *P. digitatum* stress by transcriptome data, and made the functional prediction of the related genes. These results can provide a theoretical basis for further understanding the role of CsWRKY TFs in pathogen stress and lay a foundation for future functional verification of excellent candidate genes.

## 2. Materials and Methods

### 2.1. Genome-Wide Identification of the CsWRKY TFs

Relevant data, such as *C. sinensis* genome (v3.0) sequences, protein sequences, and annotation information, were downloaded from the Citrus Pan-Genome to Breeding Database (CPBD, http://citrus.hzau.edu.cn/download.php, accessed on 25 June 2022). Combined with the WRKY Hidden Markov Model (PF03106, http://pfam.xfam.org/ accessed on 25 June 2022), a domain search of the orange genome-wide proteins was performed using the hmmsearch program to select protein sequences with E-value < 1 × 10^−5^. Secondly, homology matching of sweet orange whole protein sequences was performed using the Blastp program with 71 AtWRKY TF sequences as a reference, and those with E-value < 1 × 10^−5^ were screened. Two screens were combined and de-duplicated. Online protein domain analysis tools SMART (http://smart.embl-heidelberg.de/, accessed on 3 July 2022) and CDD (https://www.ncbi.nlm.nih.gov/Structure/cdd/wrpsb.cgi, accessed on 3 July 2022) were used to determine the presence of WRKY domains in the candidate sequences [26,27], then eliminating missing or incomplete ones, finally obtaining CsWRKY TF family members.

### 2.2. Calculation of Physicochemical Parameters of CsWRKY TFs

The numbers of amino acids, molecular weight, theoretical isoelectric point (protein-wide included), and instability coefficient of the CsWRKY TFs were calculated using the online analysis tool ProtParam (http://www.expasy.org/tools/protparam.html, accessed on 5 July 2022). We used the Plant-mPLoc tool (http://www.csbio.sjtu.edu.cn/bioinf/Cell-PLoc-2/, accessed on 5 July 2022) to predict the subcellular localization of CsWRKY sequences. The gene length, coding region length, and G-C contents in 2000 bp upstream of the coding genes were counted by TBtools [28].

### 2.3. Classification and Phylogenetic Analysis of CsWRKY TFs

The AtWRKY and CsWRKY TFs sequences were subjected to multiple sequence alignment by ClustalX2 software (version 2.0), and the results were corrected, clipped, and embellished by Jalview software (version 2.11.2) to retain 11 amino acid sequences upstream of the heptad domain and three amino acid sequences downstream of the zinc finger domain. We integrated the results and submitted them to WebLogo (http://weblogo.berkeley.edu/logo.cgi, accessed on 11 July 2022) for sequence identification while referring to the AtWRKY TFs classification methods for categorizing [16].

The domains (65–69 cross amino acid sequences) and full-length amino acid sequences of CsWRKY and AtWRKY TFs were compared using ClustalX2. The maximum likelihood tree-building software IQ-TREE and Modelfinder were chosen to select the best model to construct the phylogenetic tree, with the bootstrap parameter set to 1000 [29]. The evolutionary tree was obtained from beautification with the online iTOL (https://itol.embl.de/, accessed on 13 July 2022).

### 2.4. Analysis of the CsWRKY Gene Structures and TF Domains

Exon-intron information of the encoding genes was obtained from the sweet orange genome annotation files. The CsWRKY TF domains were predicted and annotated using the online tool MEME (http://meme-suite.org, accessed on 17 July 2022), with the number set to 10 and other parameters to default values [30]. Finally, the results were plotted using TBtools integration.

### 2.5. Analysis of the Cis-Elements of the CsWRKY Genes

To predict the cis-acting elements in the promoter, we extracted the upstream 2000 bp base sequences of the *CsWRKY* genes by TBtools. The promoter analysis was performed using the online PlantCARE (http://plantpan.itps.ncku.edu.tw/index.html, accessed on 25 July 2022), and the results were finally collated and visualized [31].

### 2.6. Chromosomal Localization, Duplicate Gene Pairs and Ka/Ks Calculations for the CsWRKY Genes

Based on the sweet orange whole genome and annotation files, the TBtools were used to extract the coordinates, length information, and genome-wide gene density information of target *CsWRKY* genes on chromosomes. The MCScanX was used to generate the gene collinearity files [32]. Additionally, the NG methods calculated the nonsynonymous changes (Ka), synonymous changes (Ks), and Ka/ks ratio of duplicated gene pairs [33].

### 2.7. Gene Ontology (GO) Annotations and Enrichment Analysis

All CsWRKY TF sequences were extracted from the sweet orange whole genome protein sequences files. The eggNOG-mapper (http://eggnog5.embl.de/#/app/home, accessed on 5 August 2022) finished functional annotation [7]. The results were manually filtered and visualized by the online plotting tool ChiPlot (https://www.chiplot.online/bar_plot_width_category.html, accessed on 6 August 2022).

### 2.8. Analysis of CsWRKY Gene Expression Pattern under Biotic Stress

#### 2.8.1. Strain Isolation, Identification and Fruit Biostress Treatment

The method of Costa et al. [34] was referred to as isolate *Penicillium* disease samples. The mature sweet orange fruits infected with *Penicillium* were collected from Xinping County, Yunnan Province, China. Potato dextrose agar (PDA) medium was selected for isolation and purification, followed by monosporial culture, and finally sent to the company for sequencing and identification. The representative *P. digitatum* strain xp3 was chosen and inoculated onto a PDA medium by a plate coating method and incubated in a constant temperature incubator at 25 °C for three days. Then, the culture dishes were rinsed twice with sterile water, and the rinsing solution was collected in a centrifuge tube and shaken well to obtain a spore suspension.

**Figure 1:**
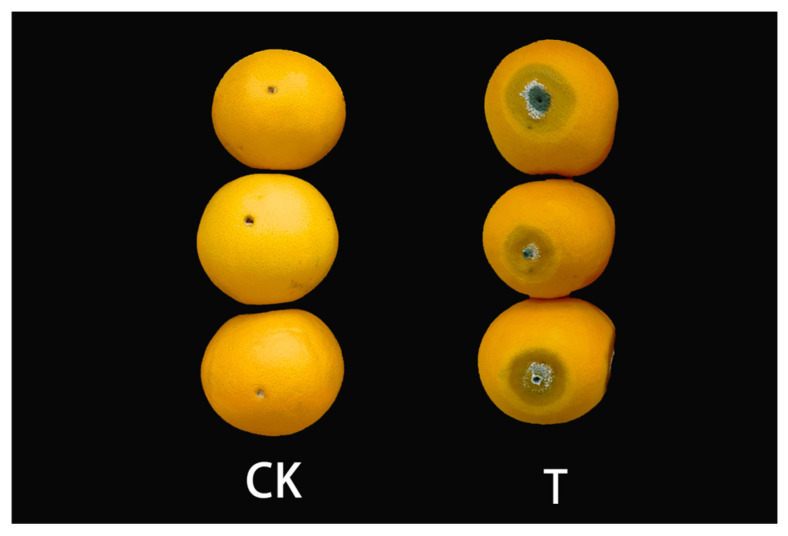
Sweet oranges infected with *P. digitatum* and controls. CK, control fruits; T, fruits infected with *P. digitatum*.

Surface disinfection of 62–67 mm mature, medium-sized fruits harvested from the same asexual line in Xinping County by treating them with 4% sodium hypochlorite solution. Then, four holes (4 mm length × 4 mm width × 3 mm depth) were punched on the equator of each fruit skin with a punching bear after autoclaving, 30 μL of spore suspension was injected into each well separately as the treatment group, while the treatment injected with an equal amount of autoclaved distilled water was used as the control, and five Bing tang oranges were treated in each treatment and control group [35]. The treated fruits were packed into sterile plastic boxes and incubated in an ultra-clean bench at 25 °C for seven days, respectively. A sample of the peel with a radius of about 3 cm was taken from each hole, quickly stored in liquid nitrogen, and sent to Bioyi Biotechnology Co., Ltd. Wuhan, China, for transcriptome sequencing. Three biological replicates were set up for both the treatment and control groups (see Figure 1).

#### 2.8.2. Expression Profiling of *CsWRKY* Genes under *P. digitatum*

To avoid many junctions and low-quality transcriptomic data, we used Trim-galore and Trimmomatic to de-join and filter low-quality data first and then used FastaQC to quality check the data to ensure that all transcriptomic data had a Q-value, more than thirty. The reads were mapped to the reference genome using hisat2. Finally, the reads were compared to the reference genome using the FeatureCounts toolkit of Rsubread software (version 2.12.2). The fragments of each gene quantified the expression of each gene. Heat maps of *CsWRKY* gene expression patterns were constructed using the R package Pheatmap based on log2^FPKM^ values [36]. Additionally, we used DESeq2 to analyze differential genes under biotic stress. Finally, genes with Fold Change ≥ 2.00 and *p*-value ≤ 0.05 were defined as significantly differentially expressed genes [37].

## 3. Results

### 3.1. Identification of CsWRKY TF Members and Calculation of Physical and Chemical Parameters

The whole sweet orange genome was scanned using BLAST alignment and the Hidden Markov Model to remove redundant sequences and sequences with short or missing domains before being further analyzed for the presence of intact WRKY domains in candidate members using the Pfam and CDD databases. Forty-seven reliable CsWRKY TFs were randomly renamed CsWRKY1-CsWRKY47 (Supplementary Table S1). It was shown that the G-C content in the promoter had a strong influence on promoter strength. The theoretical isoelectric point was a vital physicochemical property that affected the structure and function of the protein and could be used as an evolutionary marker for promoters and TFs, respectively. Both accompanied the characteristics of long-term biological evolution and showed a decreasing trend in plant cells [38]. So the effects of both on *WRKY* gene promoter intensity were explored (Table 1). The amino acid numbers and relative molecular weights of the WRKY TFs ranged from 176 (CsWRKY32) to 953 (CsWRKY29), 20.11 (CsWRKY11) to 106.56 (CsWRKY32) kDa, with mean molecular weights and mean isoelectric points of 45.23 KDa and 6.99, the instability coefficients larger than 40, subcellular localization showed all of them located in the nucleus.

In terms of evolutionary progression, promoters were more ready to turn on TFs due to reduced G-C content, and they showed a trend toward less positive charge when TFs did not require a more positive one. Altruistic interaction suggested a mutually beneficial co-evolutionary pattern between promoters and TFs in plant cells [39]. By comparing the overall mean isoelectric point (pI = 7.51) of the corresponding whole proteome, the CsWRKY TFs showed a decreasing trend, and the G-C content of the promoter region also presented an overall decreasing trend, which indicated a strong correlation between the two in the CsWRKY TFs evolution process.

### 3.2. Classification and Phylogenetic Analysis of CsWRKY TFs

**Figure 2:**
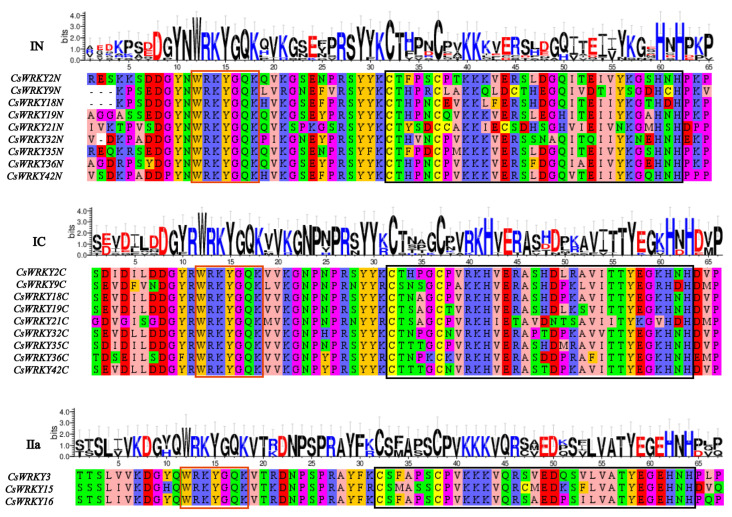

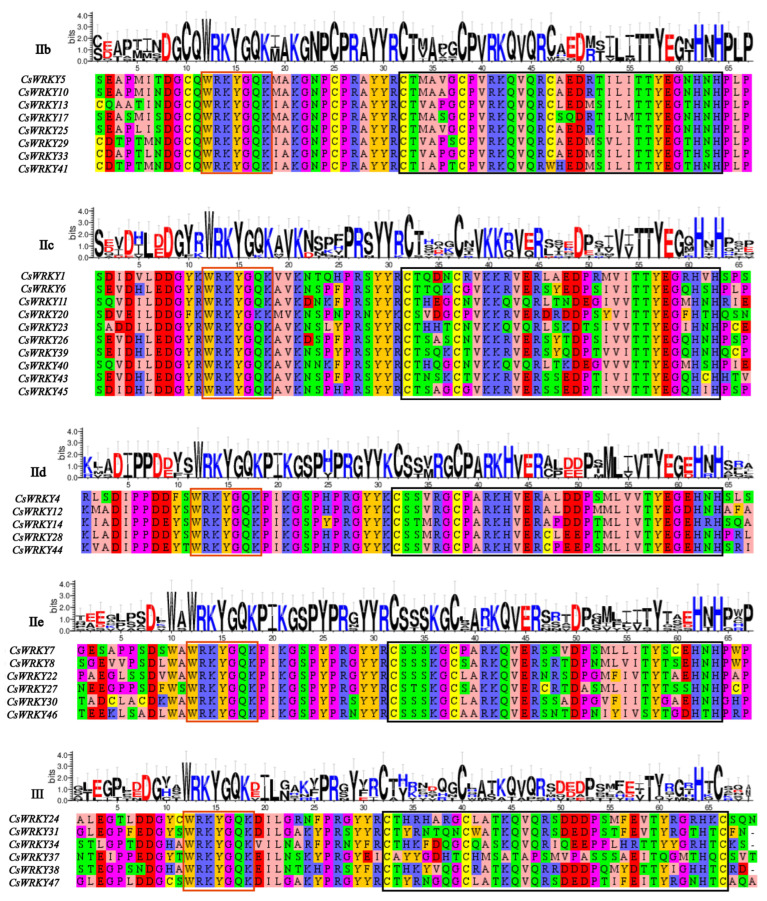
Results of multiple sequence alignment of domains of CsWRKY TFs. Heptad domains were displayed in the red box, while C2H2 or C2HC-type zinc finger structures were displayed in the black box. Roman numerals I, II, and III represented different groups. IN represented the N-terminal WRKY domain of group I, and IC represented the C-terminal WRKY domain of group I. IIa,b,c,d,e were different subgroups. The sequence logos were performed using the online mapping tool WebLogo. The *Y*-axis (in bits) indicates the overall height of the stack. The type of amino acids occurring at each position reflected the preference of the sequence at that position. The size of each letter was positively correlated with the frequency of amino acids at that position.

**Figure 3:**
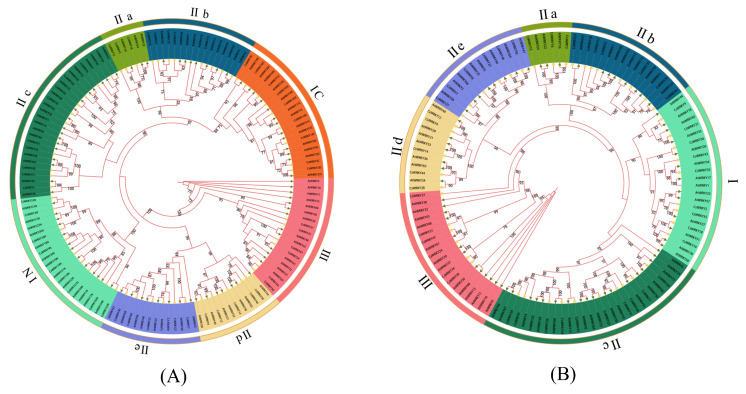
Rootless phylogenetic tree of CsWRKY and AtWRKY TFs. (**A**) A phylogenetic tree based on the domains of CsWRKY and AtWRKY TFs (65–69 spanning amino acid sequences) was constructed using the maximum likelihood tree-building software IQ-TREE, with the optimal model being JTTDCMut + I + G4. (**B**) The phylogenetic tree based on the full-length amino acid sequences of the CsWRKY and AtWRKY TFs was constructed using the maximum likelihood tree-building software IQ-TREE, with the optimal model JTTDCMut + F + R5. The calibration parameters bootstrap was repeated 1000 times and retouched by iTOL. Branches with bootstrap ≥ 70% were shown, unmarked branches represent bootstrap < 70%, and the outer Roman numerals indicate grouping.

Generally speaking, two or more genes with similar sequences had high similarity in function and regulation and vice versa. We detected the taxonomic situation and evolutionary relationships of each CsWRKY TFs by performing multiple sequence comparisons and constructing phylogenetic trees for the structural domains of CsWRKY and AtWRKY TFs (Figure 2 and Figure 3). Members of subfamily I contained two heptad domains and two C2H2-type zinc finger domains, members of subfamily II included a WRKY domain and a C2H2-type zinc finger domain, and members of subfamily III contained a heptapeptide-conserved sequence and a C2HC-type zinc finger domain. Among them, CsWRKY20 from group IIc showed the heptad domain of WRKYGKK, and similar ones, such as WRKYGRK and WSKYEQK, were also found in *Populus trichocarpa* [40], which showed that the heptad domains and zinc finger domains of CsWRKY TFs had an overall low degree of variation. The subfamilies I, II and III were further subdivided into eight subgroups by combining sequence similarities. Subfamily I contained nine CsWRKYs; subfamily II included 32 members (IIa3, IIb8, IIc10, IId5, IIe6), the largest; subfamily III had six members. At the evolutionary level, the CsWRKY and AtWRKY were evenly distributed on each branch, which indicated that they had similar evolutionary patterns. The N-terminal and C-terminal WRKY domains in subfamily I were clustered into different evolutionary parts, and the five subgroups in subfamily II were not all clustered into one. While IIa, IIb, IId, and IIe belonged to the same subgroup, respectively, which were close to each other and belonged to parallel evolutionary processes but indicated the evolutionary differences between discrepant subgroups. After comparing the two plots in Figure 3A,B, we confirmed that the evolutionary tree construction based on both domains and full-length protein sequences could accurately indicate taxonomic and phylogenetic relationships of CsWRKY TFs, achieving mutual validation support.

### 3.3. Analysis of the CsWRKY Gene Structures and TF Domains

**Figure 4:**
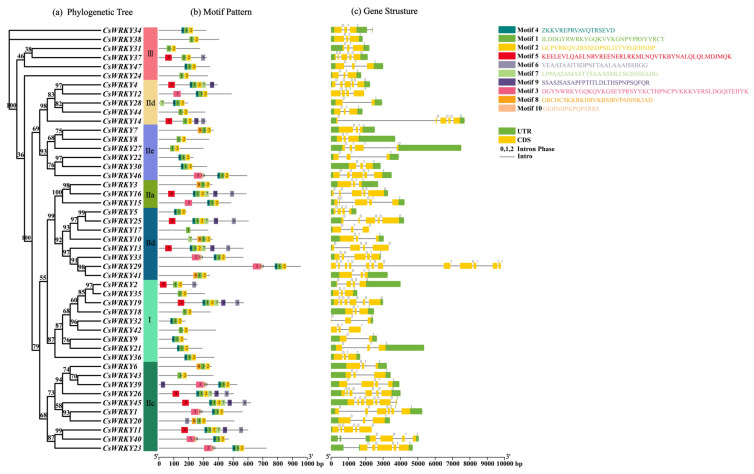
Phylogenetic relationship, motif composition, and coding gene structure of WRKY TFs. (**a**) The phylogenetic tree was constructed based on the full-length amino acid sequences of CsWRKY TFs by IQ-TREE. (**b**) The forty-seven motifs of CsWRKY TFs were predicted by the MEME. Different colored boxes and numbers indicated various motifs, and numbers 1–10 were shown correspondingly in the various colored boxes. Short sequences of various motifs have been annotated behind them. (**c**) Intron-exon structures for forty-seven genes were derived from the TBtools software. Exons, introns, and non-coding regions (UTRs) were indicated by green boxes, gray lines, and yellow boxes, respectively. The numbers 0, 1, and 2 represent the phase information of the intersection point between introns and exons.

Highly conserved gene sequences are vital for the survival of this species, and the domains have crucial functions that cannot be altered and are the core of the genes, and they may encode some important proteins [41]. Based on the phylogenetic relationships, we performed conserved domain analysis and gene structure analysis of 47 CsWRKY TFs to construct an evolutionary tree based on the full-length amino acid sequences (Figure 4). Ten motifs were mined through the web tool MEME, among which motifs 1, 2, and 3 were considered WRKY domains. Each CsWRKY contained 1 to 7 motifs, ranging from 14 to 50 bp in length, and all members except CsWRKY37 included motifs 1 and 2. Members of the same subfamily (subgroup) of CsWRKY TFs had similar motif composition and arrangement. Some motifs presented specifically or simultaneously in multiple evolutionary branches, e.g., members of the group I contained motifs 1, 2, 3, 4, and 10, and motifs 7 and 9 were specific to subgroup IIb. Motif 8 was only to subgroup IId, and motif 6 was presented in subgroups IIa, IIb, and IIe, related to the functional differences. We found that the coding sequences corresponding to the WRKY domain all contained an intron with a highly conserved position, but the significance of their existence was unclear.

To provide more insight into the relationship between the coding gene structure and phylogeny, the exon-intron of the *CsWRKY* genes was demonstrated by TBtools. The forty-seven *CsWRKY* genes contained 2 to 12 exons and 1 to 11 introns, with high variation in number. In another example, twenty-two genes had three exons, seven genes had four exons, nine genes had five exons, and one gene had twelve exons, with CsWRKY29 having the highest number of exons and introns, which to some extent led to the functional diversity. Genes within the same subfamily (subgroup) usually had a similar number of exons, e.g., members I and III mostly contained three exons and two introns, while the number varied widely among different subgroups.

Here, synthesizing the structural similarities and differences in TFs on the same or different branches and groupings, it can be seen that the *CsWRKY* genes structure and conserved domain results exhibit a strong correlation with phylogenetic relationships, which can support the reliability of the CsWRKY TF family classification.

### 3.4. Analysis of Cis-Acting Elements of the CsWRKY Gene’s Promoter

**Figure 5:**
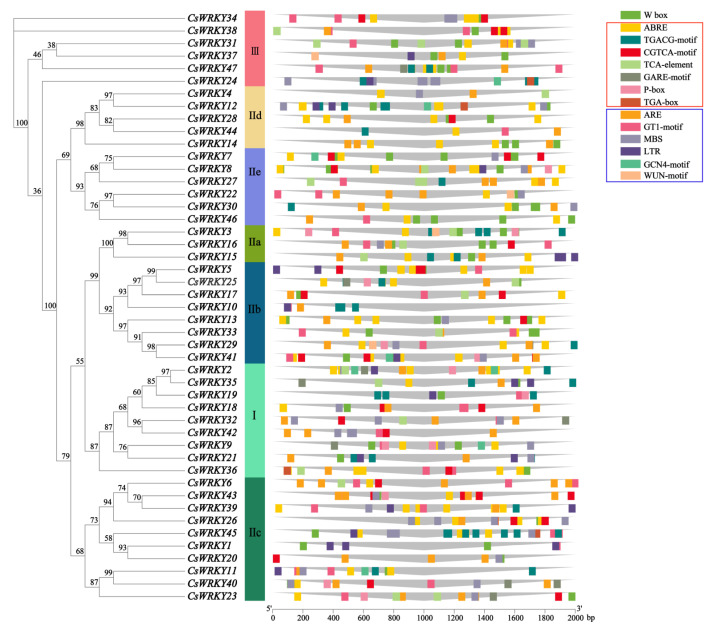
Cis-acting elements in the promoter of *CsWRKY* genes. The distribution of cis-acting elements in the upstream 2000 bp region of 47 *CsWRKY* genes was depicted. Seven element types in the red box were plant-induced response regulatory elements, and the six types in the blue box were stress response-related elements, with each type of element sorted from the highest to the lowest number. Different components were indicated by various shapes and colors.

Plant cis-acting elements played a vital role in the global regulation of gene expression. After a cis-acting element analysis of the 2000 bp upstream region of forty-seven *CsWRKY* genes by the PlantCARE, 14 common cis-acting elements were mainly predicted (Supplementary Table S2), as well an evolutionary tree was constructed based on the full-length sequences of CsWRKY TFs (Figure 5). Including the W-box binding site, seven regulatory elements involved in phytohormone-induced responses (ABRE, cis-acting element involved in the abscisic acid responsiveness; CGTCA-motif and TGACG-motif, cis-acting regulatory element involved in the Me-JA-responsiveness; TCA-element, cis-acting element involved in salicylic acid responsiveness; P-box and GARE-motif, gibberellin-responsive element; TGA-box, part of an auxin-responsive element) and six stress-related promoter regulators (ARE, a cis-acting regulatory essential for the anaerobic induction; GT1-motif, light responsive element; MBS, MYB binding site involved in drought-inducibility; LTR, cis-acting element involved in low-temperature responsiveness; GCN4_motif, cis-regulatory element involved in endosperm expression; WUN-motif, wound-responsive element). All forty-seven *CsWRKY* genes contained 5 to 32 common acting elements, with the most frequent being ABRE and the least being WUN-motif, with up to 32 predicted ones in *CsWRKY45*.

Notably, forty-seven *CsWRKY* genes predicted 73 W-boxes, suggesting that specific binding of the WRKY domain to the W-box was essential for coordinated transcriptional activation. *CsWRKY45* contains 18 regulatory elements associated with the Me-JA response. *CsWRKY2* had ten regulatory factors related to abscisic acid response. Nearly half of the ratio is mainly to biological processes such as hormone signaling under specific adversity stresses.

### 3.5. Chromosome Distribution, Gene Density and Duplication, Collinearity Analysis, and Ka/Ks Calculation for CsWRKY Genes

**Figure 6:**
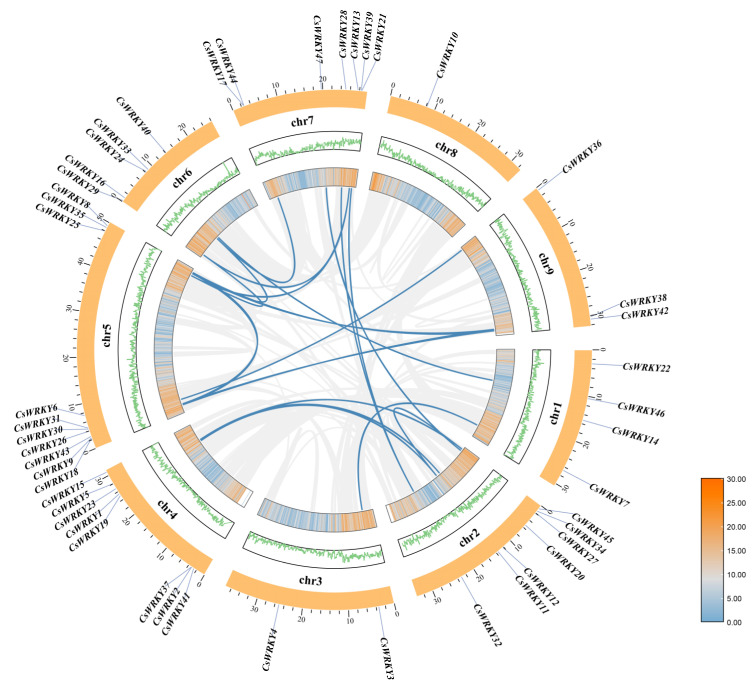
Chromosome distribution, gene density, gene duplication, and collinearity analysis for *CsWRKY* genes. The gray line indicated all duplicated genes in the sweet orange genome, blue presents duplicated *WRKY* gene pairs, the gene density was shown as a heat map, and line graph with increasing density from blue to gray to yellow, and chromosome names were shown on the outside of each chromosome.

To understand the distribution and genome-wide density of *CsWRKY* genes on sweet orange chromosomes, we localized 47 *CsWRKY* genes on nine chromosomes of the sweet orange genome by TBtools (Figure 6). There were 4, 7, 2, 8, 10, 5, 7, 1, and 3 *CsWRKY* genes localized on chromosomes 1, 2, 3, 4, 5, 6, 7, 8, and 9, respectively, with an overall uneven distribution and no significant correlation between chromosome length and the number of *CsWRKY* genes. However, the whole genes were relatively evenly distributed on each chromosome.

Genome-wide duplication events are a common phenomenon in biology and currently reported in many species. Forty-seven *CsWRKY* genes duplication events were analyzed by MCScanX, consisting of 21 pairs of duplication genes (*CsWRKY7*-*CsWRKY3*, *CsWRKY8*-*CsWRKY43*, *CsWRKY8*-*CsWRKY24*, *CsWRKY8*-*CsWRKY42*, *CsWRKY11*-*CsWRKY45*, *CsWRKY11*-*CsWRKY19*, *CsWRKY12*-*CsWRKY1*, *CsWRKY16*-*CsWRKY25*, *CsWRKY16*-*CsWRKY13*, *CsWRKY18*-*CsWRKY35*, *CsWRKY18*-*CsWRKY38*, *CsWRKY24*-*CsWRKY44*, *CsWRKY25*-*CsWRKY13*, *CsWRKY30*-*CsWRKY36*, *CsWRKY32*-*CsWRKY21*, *CsWRKY32*-*CsWRKY28*, *CsWRKY34*-*CsWRKY47*, *CsWRKY35*-*CsWRKY38*, *CsWRKY43*-*CsWRKY42*, *CsWRKY45*-*CsWRKY19*, *CsWRKY46*-*CsWRKY33*), with 27 *CsWRKY* genes exhibiting fragment duplication among them. These duplicated fragments possibly influenced the number of WRKY TFs in the sweet orange genome and their distribution on chromosomes. The functional differentiation of duplicated gene pairs was a source for the new genes generated in plants, promoting the expansion of the CsWRKY TF family and injecting new impetus into plant genome evolution. However, evolutionary patterns and mechanisms of duplicated genes were not very clear.

Changes in amino acids maybe lead to radical changes in their function and conformation, resulting in an advantage or disadvantage of evolutionary selection [42]. To understand the evolutionary selection of the duplicated homologous *CsWRKY* genes subjected during evolution and whether this selection contributed to the organism’s fitness, we calculated the ratio between nonsynonymous and synonymous substitutions (Ka/Ks) for twenty-one pairs of duplicated genes (Supplementary Table S3). It was shown that genes were subject to positive selection for the Ka/Ks ratio > 1, neutral selection for the ratio = 1, and purifying one for the ratio < 1 [27]. Twenty-one pairs of duplicated genes were identified, and 90.5% had Ka/Ks values < 1, suggesting that *CsWRKY* genes in sweet orange may have been subject to purifying selection pressure during speciation, while 9.5% of duplicated gene pairs had zero Ks, which may have resulted from single-base mutations.

### 3.6. Gene Ontology (GO) Enrichment Analysis

**Figure 7:**
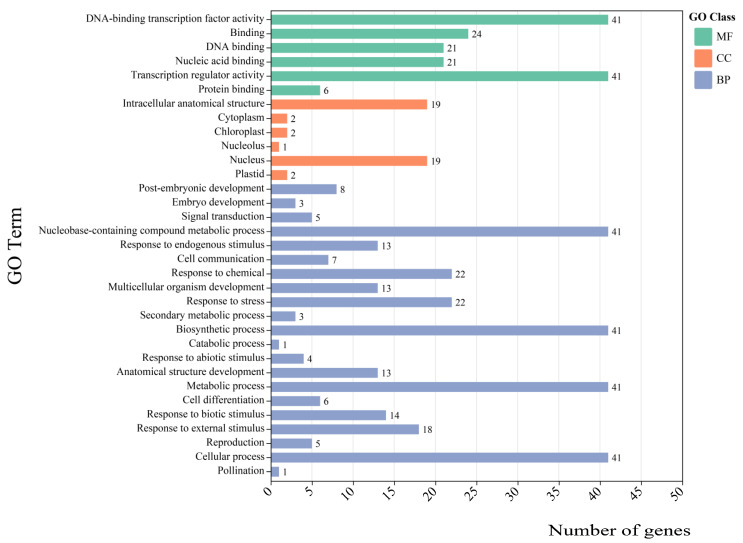
GO annotation results of *CsWRKY* genes GO-Level 3. The results were divided into three major categories: molecular functions, cellular components, and biological processes, shown in green, yellow and blue, respectively. The *x*-axis indicates gene functions, and the *y*-axis indicates the partial GO Term.

Genome sequencing data have shown that most genes with core biological functions are common to all eukaryotes. GO annotation classification system is subject to elaborate biological macromolecules with three major underlying classifications: cellular components, molecular function, and biological processes [43]. To further understand the expression of forty-seven *CsWRKY* genes, we performed GO annotation to analyze (Supplementary Table S4). Forty-one of forty-seven *CsWRKY* genes were up to 387 GO terms, mainly including three categories, of which 87.86% were to biological processes. Since nearly 400 GO terms were done, we selected 41 GO-Level 3 for display (Figure 7). The molecular functional categories mainly included heterocyclic compound binding (GO:1901363) and DNA-binding transcription factor activity (GO:0003700). Intracellular anatomical structure (GO:0005622) and membrane-enclosed lumen (GO:0031974) were in the cellular components. The category of biological processes was mainly involved in the organic substance metabolic process (GO:0071704), regulation of the process (GO:0050789), nitrogen compound metabolic process (GO:0006807), cellular metabolic process (GO:0044237), and biosynthetic process (GO:0009058).

Most disease-resistance genes interacted with the TFs to enhance disease resistance. For example, *Xa1* shear in vivo released an intracellular kinase domain that translocates to the nucleus and interacts with the OsWRKY62 TFs to enhance rice resistance to leaf blight [44]. Pita encoding product interacted with the expression product of AVR-Pita, a non-virulent gene of rice blast fungus, to trigger a disease-resistance response [45]. These disease resistance genes were to the essential functions of the molecule and processes related to cells, tissues, organs, or living organisms, and gene product localization.

### 3.7. The Transcript-Level Analysis of CsWRKY Genes under Biotic Stress

**Figure 8:**
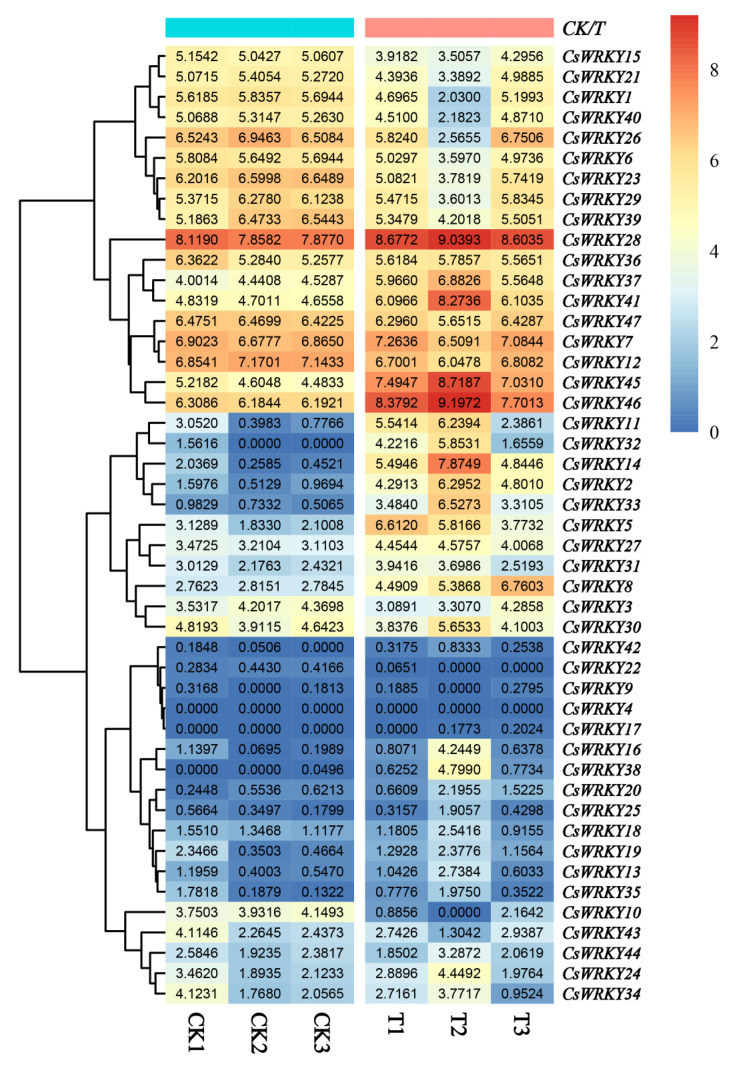
Heat map of forty-seven *CsWRKY* gene expression profiles under *P. digitatum* stress. Different colored boxes represented different log2^(FPKM)^ values, and the expression gradually increased from blue to white to red. CK represented the control group, and T represented the treatment group.

To investigate the potential role of *CsWRKY* genes under biotic stress, we analyzed the transcriptome data of sweet oranges after five days of infection by *P. digitatum* and obtained the expression patterns of 47 *CsWRKY* genes in sweet oranges (Figure 8). We screened thirteen differentially expressed genes (value > 2), including *CsWRKY2*, *CsWRKY5*, *CsWRKY8*, *CsWRKY11*, *CsWRKY14*, *CsWRKY20*, *CsWRKY23*, *CsWRKY27*, *CsWRKY28*, *CsWRKY31*, *CsWRKY37*, *CsWRKY45*, and *CsWRKY46*, only *CsWRKY23* was a down-regulated expression gene, while the rest were all up-regulated expression genes. Notably, *CsWRKY2* and *CsWRKY14* expression were up-regulated 67 and 155-fold, all indicating that different copies of different genes had different expression patterns in sweet orange under biotic stress. *CsWRKYs* were extensively involved in the growth and development of sweet oranges in response to *P. digitatum* infection. Interestingly, *CsWRKY17* and *CsWRKY32* did not express in ripe fruits but did significantly after pathogen infection treatment.

In *Arabidopsis*, the functions of most WRKY TFs were verified and widely involved in plants’ response to pathogen infections. We selected six highly expressed *CsWRKYs* representative genes for functional inference based on the evolutionary relationship and structural similarity between CsWRKY and AtWRKY TFs (Table 2). Combined with the motif prediction results, we found that the N and C-terminal ends of the highly expressed differential genes contained a novel motif5 and motif6. Respectively, those present might promote rapid expression. The promoter region had abundant TGACG-motif, W-box core promoter elements that responded to pathogen infection and bound to disease-resistant genes, and then activated their transcription in response to pathogen infection. The expression level of the silenced *CsWRKY32* was altered after *P. digitatum* infection, and a vital motif4 was predicted in front of the W-box, a fungus-inducible responding element Me-JA (CGTCA-motif), which to some extent conferred a structural basis for resistance to the pathogen.

## 4. Discussion

WRKY family is a prime class of transcription factors in plants involved in the plant response to biological and abiotic stress regulation. Meanwhile, many *WRKY* genes in the genomes of higher plants also indicate that they play a broad role in development and evolution. This class of genes has been studied in monocotyledons and dicots, mainly due to the increasingly mature genome-wide, transcriptome sequencing technologies, bioinformatics tools, and the mature research systems for identification, classification, and biological functions of model plants, such as *A. thaliana* and *G. max*, which lay the foundation for the study of the WRKY TFs family in non-model species. Previous studies on *CsWRKY* genes focused on the differences in expression patterns of stems, leaves, fruits, or roots at different developmental periods and the analysis of expression under abiotic stress such as drought, cold, and high salt, as well as mechanistic studies. However, systematic studies on WRKY TFs in sweet orange under *P. digitatum* stressing have rare reports.

In this study, 47 CsWRKY TFs were identified by the genome-wide analysis, which was close to the woody plants, such as *Zanthoxylum bungeanum* (38 ZbWRKY) [52] and *Prunus dulcis* (62 PdWRKY) [53]. The number differed significantly compared to herbaceous plants such as *Chenopodium quinoa* (92 CqWRKY) [54] and *Oryza nivara* (97 OnWRKY) [55], which indirectly indicated that monocotyledonous genomes might be more abundant than dicotyledonous genomes on the whole [39]. In similar studies, 51 CsWRKY TFs were identified by Ayadi, and nine members were not located on chromosomes [56], compared with the 47 TFs we identified located on nine chromosomes. Our identification results may be more accurate than Ayadi’s study and can better present information about the members of the CsWRKY TF families. As we used the latest draft genome of sweet orange (*Citrus sinensis* v3.0), the method used was easier to understand, and the screening criteria were much more rigorous. Sweet oranges often grow in hilly and poorly established environments, though it does not contain many WRKY TFs, and we assume that the expression and transcriptional regulation of most *WRKY* genes is vital for responding to various stresses. Many small-scale gene duplications or doubling events have occasionally occurred in woody or herbaceous plants during their long evolutionary history, which can easily lead to unequal gene numbers in different species. The duplication of genes in these regions has little effect on conserved sequences and ensures the exercise of TFs function [2]. Various proteins interact with WRKY TFs, and the vital WRKY TF proteins may provide important insights into their regulation and mode of action, closely related to their physicochemical properties. The G and C content of the promoter regions and the isoelectric point of their coding proteins determine the timing, intensity, and location of regulated gene expression. Forty-seven *CsWRKY* gene promoters showed decreasing trends in G and C content and isoelectric points of TFs, consistent with the co-evolutionary pattern of promoters and transcription factors in the plant kingdom [38].

During the visualization of the CsWRKY domain, novel heptad domains such as WRKYGKK were identified, which may have resulted from single base mutations. It has been reported that single or multiple amino acid changes in tobacco reduce the affinity for binding to the W-box and even alter its function [57]. Phylogenetic relationships showed that forty-seven CsWRKY TFs were divided into eight subgroups, with the IIc subgroup having the most members and the IIa subgroup the least, which was verified in almost all reported WRKY TFs [8,10,35,53].

Members of the same subfamily on the phylogenetic tree had the same or similar intron-exon structure, domain type, and arrangement order [58]. We observed some unique or missing domains, introns, and exons in some groups. The CsWRKY TF families underwent intron-exon losing or incoming events during the evolutionary process, and these differences may be related to *CsWRKY* gene functional diversity [59]. In *Arabidopsis*, the AtWRKY27-positive regulator was essential for defense against dead body trophic *B. cinerea* [60]. The intron-exon structure of the highly expressed *CsWRKY2* was highly similar to that of *AtWRKY27*. However, the domains were arranged in a different order, probably due to differences in the domain arrangement between various species at different evolutionary levels. The expression levels of *AtWRKY11* and *AtWRKY17* were up-regulated at different developmental stages under ABA, salt, and osmotic stress [61]. The highly expressed gene *CsWRKY14* in sweet orange was highly homologous to the above genes, but they played different roles in response to various stresses, and whether the *CsWRKY14* could be highly expressed under abiotic stress remained to be verified. It has been shown that the domains and gene structures exhibit strong correlations with phylogenetic relationships and biological functions. However, only the functional validation of very few members of TF families had been accomplished in most species, so the overall realization of evolutionary relationships and taxonomic studies by gene functions have not been reported in non-model species, which deserves further research.

WRKY TFs bond to target gene promoter W-box cis-acting elements and participate in autoregulation or cross-regulation by regulating multiple stress signaling pathways to activate, enhance, or repress the expression of target genes [62]. Seventy-three W-Box binding sites were identified from 47 CsWRKY TFs, even as many as 4 in CsWRKY7 in this paper. There were 22 CsWRKY TFs with multiple W-Box binding sites (value >2). However, for the 56 CsWRKYs with much W-boxes identification, the percentage was 33.11% in the tea trees [8]. In barley, the HvWRKY38 TF was associated with two adjacent W-boxes to activate the transcription of downstream target genes effectively [63], fully demonstrating the vital regulatory role of W-boxes in plant response to biotic or abiotic stress. When ABA and Me-JA accumulated to a certain level in plants, they would initiate the expression of disease-resistant defense genes, prompting a defense response and thus exhibiting strong resistance to disease [64]. We predicted abundant hormone-regulated elements (154 ABRE elements and 85 TGACG-motif73), suggesting many inducible promoters with cumulative effects on the expression of downstream genes in addition to the required ones such as W-box elements, which was similar to the predicted promoters 2000 bp upstream of WRKY genes in pineapple [11] and watermelon [26]. In this study, we found that CsWRKY2 contained many listed vital promoter elements, indicating that this gene responds to P. digitatum infection as a process in which multiple cis-acting genes interact in an integrated manner. The CsWRKY14 contained only ABRE, ARE, and W-box elements. It indicated a difference in the mechanism of action of the two genes in response to fungal infection. It is inferred that these promoter elements may respond to pathogen stress, drought stress, high-temperature stress, low-temperature stress, salt stress, and plant damage stress.

Genome-wide duplication events are a common phenomenon in biology; gene duplication events are a dominant force in the evolution of plant genomes and duplicated gene pairs are now introduced in many species providing the molecular basis for species adaptation to different environments and biodiversity [65]. Among the 47 *CsWRKY* genes, there were 21 sets of fragment duplication genes, three duplications (*CsWRKY24*, *CsWRKY42*, and *CsWRKY43*) were amplified from the *CsWRKY8* gene alone, and the duplication events were very conserved, with the number of fragment duplications far exceeding the number of tandem duplications, indicating that fragment duplication events were one of the main drivers of the evolution of the CsWRKY TFs. We noted that among the 21 pairs of duplicated genes, most of them are distributed in regions with high gene density, and chromosome 5 had the highest number of duplicated genes and the most distributed *CsWRKY* members, speculating that there was rich genetic diversity on chromosome 5, which may have evolutionarily created and preserved a wide variety of variant types. The Ka/Ks ratios of 21 pairs of duplicated genes showed that 90.5% of the Ka/Ks values were < 1. It inferred that *CsWRKY* genes might have been subjected to purify selective pressure during speciation, which meant that in most cases, the *WRKY* genes were selected to eliminate harmful mutations and keep the protein unchanged while maintaining its original function.

In the results of phylogenetic tree analysis, different subgroups had some preferences in response to various biotic or abiotic stress, while members from the same evolutionary branch often had many similarities in stress functions. For example, some WRKY from subfamilies IIa [66] and III [51] were mainly involved in regulating leaf senescence in response to environmental stress, and some *WRKY* genes from subfamily I [46] and subgroup IIb [67] were involved in response to fungal and bacterial pathogens. Since highly expressed genes usually play a vital role in plant development, *CsWRKY* genes can be expressed strongly and rapidly under specific biotic stress. In the present study, *CsWRKY2* from group I and *CsWRKY14* from group IId were up-regulated significantly in response to *P. digitatum* infection, and *CsWRKY14* from subgroup IId and *CsWRKY46* from subgroup IIe were identified as differentially expressed genes, both likely involved in the defense response to *P. digitatum* infection. These highly and differentially expressed genes *WRKY2*, *5*, *8*, *11*, *14*, *20*, *23*, *27*, *28*, *31*, *37*, *45*, *46* were essential for sweet orange growth and were good candidates for future functional analysis. *CsWRKY17* and *CsWRKY32* were not expressed in mature fruits but did after *P. digitatum* infection, so the two genes were also listed as valuable candidates.

The function prediction employing *AtWRKY* genes with similar structures to fifteen *CsWRKY* genes as a reference showed that some genes might act individually in response to multiple stress, depending on the signaling pathways and many phytohormone signaling cascades in plant stress responses. *CsWRKY11* and *CsWRKY45*, the only pair of duplicated genes among the differentially expressed genes, may be involved in leaf senescence and defense against *Pseudomonas syringae* responses. However, the difference in expression was nearly one-fold, which was consistent with Wendel’s [68] genomic evolutionary pattern for polyploids, i.e., two duplicated genes in functional divergence may take a long time to occur but diversity in expression can start to occur within a short period of inches after the duplication.

## 5. Conclusions

In this study, 47 different CsWRKY TFs were identified and classified into eight subgroups (three subfamilies), and their expression patterns were analyzed. The exon-intron structures and domains of the *CsWRKY* genes strongly supported the classification results. Fourteen induced-response and stress-response elements were also identified in the promoter regions. Expression profiles indicated that forty-five genes involve in resistance to fungus infection. Additionally, fifteen significantly differentially expressed genes were essential for sweet oranges in response to *P. digitatum* infection, and their response varied with the degree of stress. In summary, this study provides a solid basis for studying the regulatory mechanisms of WRKY TFs in response to pathogen infection in sweet orange, and the results will help provide insights for further exploration of the role of CsWRKY TFs in biotic stress response.

## Figures and Tables

**Table 1:** Analysis of physicochemical properties of CsWRKY TFs.

Sequence ID	Rename ID	GC Content (%)	CDS Length (bp)	Number of Amino Acid (aa)	Molecular Weight (kDa)	Theoretical pI	Instability Index	Subcellular Localization
*Cs_ont_4g020050.1*	*CsWRKY1*	0.3485	1692	563	60.9	6.58	44.02	Nucleus
*Cs_ont_4g001570.1*	*CsWRKY2*	22.00%	777	258	28.81	8.94	46.67	Nucleus
*Cs_ont_3g005460.1*	*CsWRKY3*	26.35%	1077	358	38.69	9.3	49.17	Nucleus
*Cs_ont_3g025790.1*	*CsWRKY4*	26.90%	1185	394	44.09	5.83	47.46	Nucleus
*Cs_ont_4g023640.1*	*CsWRKY5*	33.40%	552	183	21.1	6.09	40.61	Nucleus
*Cs_ont_5g011420.3*	*CsWRKY6*	41.00%	1059	352	39.47	9.69	58.6	Nucleus
*Cs_ont_1g024520.1*	*CsWRKY7*	29.75%	1098	365	40.41	9.26	50.83	Nucleus
*Cs_ont_5g049260.1*	*CsWRKY8*	31.60%	1011	336	37.64	5.2	58	Nucleus
*Cs_ont_5g003770.1*	*CsWRKY9*	28.80%	567	188	21.58	9.3	50.41	Nucleus
*Cs_ont_8g010340.1*	*CsWRKY10*	26.55%	1080	359	40.54	9.83	60.18	Nucleus
*Cs_ont_2g018890.1*	*CsWRKY11*	27.95%	1800	599	65.17	6.28	49.13	Nucleus
*Cs_ont_2g017490.1*	*CsWRKY12*	33.45%	1467	488	53.31	6.27	41.99	Nucleus
*Cs_ont_7g025980.1*	*CsWRKY13*	27.55%	1704	567	62.79	6.8	51.53	Nucleus
*Cs_ont_1g011130.1*	*CsWRKY14*	27.85%	960	319	35.43	8.35	45.14	Nucleus
*Cs_ont_4g027990.3*	*CsWRKY15*	35.25%	1458	485	53.28	6.18	61.61	Nucleus
*Cs_ont_6g001560.1*	*CsWRKY16*	30.70%	1767	588	63.58	6.16	47.29	Nucleus
*Cs_ont_7g003240.1*	*CsWRKY17*	27.55%	990	329	36.07	5.86	63.22	Nucleus
*Cs_ont_5g003720.1*	*CsWRKY18*	34.75%	1041	346	37.78	6.32	57.68	Nucleus
*Cs_ont_4g019270.1*	*CsWRKY19*	26.55%	1710	569	62.67	6.71	44.48	Nucleus
*Cs_ont_2g008680.1*	*CsWRKY20*	28.55%	1518	505	54.38	5.6	55.95	Nucleus
*Cs_ont_7g027310.6*	*CsWRKY21*	26.45%	876	291	32.05	6.19	60.28	Nucleus
*Cs_ont_1g003000.1*	*CsWRKY22*	29.75%	693	230	25.81	9.22	52.71	Nucleus
*Cs_ont_4g021820.2*	*CsWRKY23*	36.30%	2172	723	78.03	5.72	55.73	Nucleus
*Cs_ont_6g012890.1*	*CsWRKY24*	26.55%	987	328	36.79	5.91	57.67	Nucleus
*Cs_ont_5g047350.2*	*CsWRKY25*	32.95%	1809	602	65.19	6.02	53.49	Nucleus
*Cs_ont_5g006060.2*	*CsWRKY26*	36.45%	1506	501	54.41	8.58	49.48	Nucleus
*Cs_ont_2g003440.1*	*CsWRKY27*	33.30%	891	296	33.77	5.52	68.34	Nucleus
*Cs_ont_7g021850.1*	*CsWRKY28*	26.45%	585	194	21.8	9.25	38.53	Nucleus
*Cs_ont_6g000450.1*	*CsWRKY29*	30.00%	2862	953	106.57	5.89	52.12	Nucleus
*Cs_ont_5g006680.1*	*CsWRKY30*	35.50%	969	322	35.54	8.56	54.88	Nucleus
*Cs_ont_5g006960.1*	*CsWRKY31*	32.70%	825	274	31.08	5.12	56.04	Nucleus
*Cs_ont_2g024430.1*	*CsWRKY32*	27.85%	531	176	20.11	9.35	42.88	Nucleus
*Cs_ont_6g013730.1*	*CsWRKY33*	31.25%	1701	566	62.49	6.29	55.13	Nucleus
*Cs_ont_2g002700.1*	*CsWRKY34*	36.55%	954	317	35.13	6.8	58.73	Nucleus
*Cs_ont_5g048610.1*	*CsWRKY35*	35.00%	924	307	34.27	5.93	56.52	Nucleus
*Cs_ont_9g001150.1*	*CsWRKY36*	28.45%	1116	371	40.91	6	59.27	Nucleus
*Cs_ont_4g001580.1*	*CsWRKY37*	26.00%	966	321	35.9	7.15	49.73	Nucleus
*Cs_ont_9g026650.1*	*CsWRKY38*	33.80%	1212	403	44.53	5.06	67.94	Nucleus
*Cs_ont_7g027040.1*	*CsWRKY39*	32.90%	1578	525	56.81	6.96	49.03	Nucleus
*Cs_ont_6g019030.1*	*CsWRKY40*	32.80%	1407	468	51.88	8.67	47.78	Nucleus
*Cs_ont_4g000830.1*	*CsWRKY41*	28.85%	1026	341	36.74	9.54	44.68	Nucleus
*Cs_ont_9g027190.1*	*CsWRKY42*	29.20%	1149	382	42.24	5.55	56.49	Nucleus
*Cs_ont_5g004320.1*	*CsWRKY43*	31.70%	1095	364	41.11	5.23	55.05	Nucleus
*Cs_ont_7g003250.1*	*CsWRKY44*	26.40%	930	309	35	6.01	61.95	Nucleus
*Cs_ont_2g001540.1*	*CsWRKY45*	30.10%	1857	618	67.18	5.99	41.18	Nucleus
*Cs_ont_1g008000.1*	*CsWRKY46*	31.15%	1779	592	64.65	7.08	55.14	Nucleus
*Cs_ont_7g015280.1*	*CsWRKY47*	30.45%	1029	342	38.27	6.6	63.75	Nucleus

**Table 2:** The functional inference of *CsWRKY* genes differentially expressed in sweet orange.

Differentially Expressed Genes	Arabidopsis Gene id	Classify	Regulation Mechanism	Subcellular Localization	Major Function
*CsWRKY2*	*AtWRKY33*	I	Positive regulation	Chloroplast	Expression of *AtWRKY33* could be induced by parasitic plants, avirulent, necrotrophic fungal pathogens (*Alternaria alternata*, *Sclerotinia sclerotiorum*), and PAMPs (flg22 polypeptide, chitin, oxidative stress). *AtWRKY33* loss-of-function mutants exhibit enhanced susceptibility to *B. cinerea* [46].
*CsWRKY32*	AtWRKY3	I	Positive regulation	Nucleus	*AtWRKY3* gene expression was up-regulated significantly under salt and Me-JA stress, and *AtWRKY3* deletion affected the ROS scavenging pathway and reduced stress tolerance. Overexpression of *AtWRKY3* significantly increased plant susceptibility to bacterial pathogens [47].
*CsWRKY11*	AtWRKY45, AtWRKY72, AtWRKY22	IIc	Positive regulation	Nucleus	*AtWRKY22* mediated the senescence signaling pathway and regulated the expression of senescence-related genes. Under hypoxic (flooding) stress, the encoded genes were expressed rapidly and strongly to enhance resistance to *P. syringae* [48].
*CsWRKY45*	*AtWRKY48*	IIc	Negative regulation	Nucleus	*AtWRKY48* can be rapidly induced by *P. syringae* infection and specifically binding to the W-box sequence. JA and ET signaling pathways were negatively regulated in stress and pathogenesis-induced AtWRKY48 expression [49].
*CsWRKY14*	*AtWRKY7*	IId	Negative regulation	Nucleus	*AtWRKY7* bonds to the bZIP28 promoter via a W-box element. *AtWRKY7* mutant accumulates more bZIP28 TFs in response to Flg22, a highly conserved polypeptide at the N-terminal end of the bacterial flagellin, and the higher the degree of mutation, the higher the resistance to *P. syringae* pathovar tomato pathogenic (Pst) [50].
*CsWRKY37*	*AtWRKY70*	III	Negative regulation	Nucleus	*AtWRKY70* was constitutively expressed at all leaf developmental stages, with the highest signal intensity in senescent leaves; single mutants showed more resistance to *P. syringae*, while double mutants were more sensitive [51].

## Data Availability

The *C. sinensis* genome (v3.0) and annotation files of sweet orange are openly available in the CPBD: Citrus Pan-genome to Breeding Database (http://citrus.hzau.edu.cn/download.php, accessed on 25 June 2022). RNA-Seq data under biotic stress can be found with accession number PRJNA855348. The RNA-Seq data are publicly available at the National Center for Biotechnology Information. The other data presented in this study are available in Supplementary Materials.

## Supplementary Materials

The following supporting information can be downloaded at: https://www.mdpi.com/article/10.3390/cimb45020082/s1, Table S1: The information on the WRKY TFs family is in sweet orange; Table S2: Information on cis-elements detected in the promoter regions of the WRKY genes; Table S3: The Ka/Ks ratio of 21 pairs of tandem repeats in the sweet orange WRKY TFs family; Table S4: The GO enrichment analysis of the WRKY genes.

## References

[1] Schneider V.K., da Silva Ferrara T.F., Rocha S.V., Santos-Júnior C.D., Neo-Justino D.M., da Cunha A.F., de Oliveira da Silva J.P.M., Dos Santos Tersariol I.L., Carmona A.K., Henrique-Silva F. (2020). Recombinant expression, characterization and phylogenetic studies of novels cystatins-like proteins of sweet orange (*Citrus sinensis*) and clementine (*Citrus clementina*). Int. J. Biol. Macromol..

[2] Feng G., Li X., Wang W., Deng L., Zeng K. (2020). Effects of Peptide Thanatin on the Growth and Transcriptome of *Penicillium digitatum*. Front. Microbiol..

[3] Ulker B., Somssich I.E. (2004). WRKY transcription factors: From DNA binding towards biological function. Curr. Opin. Plant Biol..

[4] Chen F., Hu Y., Vannozzi A., Wu K., Cai H., Qin Y., Mullis A., Lin Z., Zhang L. (2017). The WRKY transcription factor family in model plants and crops. Crit. Rev. Plant Sci..

[5] Amorim L., da Fonseca Dos Santos R., Neto J., Guida-Santos M., Crovella S., Benko-Iseppon A.M. (2017). Transcription factors involved in plant resistance to pathogens. Curr. Protein Pept. Sci..

[6] Li Y., Li X., Wei J., Cai K., Zhang H., Ge L., Ren Z., Zhao C., Zhao X. (2021). Genome-Wide Identification and Analysis of the WRKY Gene Family and Cold Stress Response in *Acer truncatum*. Genes.

[7] Nan H., Lin Y., Liu J., Huang H., Li W., Gao L. (2020). Genome-wide analysis of the *WRKY* transcription factor gene family and their response to salt stress in rubber tree. Trop. Plant Biol..

[8] Wang P., Yue C., Chen D., Zheng Y., Zhang Q., Yang J., Ye N. (2019). Genome-wide identification of WRKY family genes and their response to abiotic stresses in tea plant (*Camellia sinensis*). Genes Genom..

[9] Jia C., Wang Z., Wang J., Miao H., Zhang J., Xu B., Liu J., Jin Z., Liu J. (2022). Genome-wide analysis of the banana WRKY transcription factor gene family closely related to fruit ripening and stress. Plants.

[10] Tripathi P., Rabara R.C., Langum T.J., Boken A.K., Rushton D.L., Boomsma D.D., Rinerson C.I., Rabara J., Reese R.N., Chen X. (2012). The WRKY transcription factor family in *Brachypodium distachyon*. BMC Genom..

[11] Xie T., Chen C., Li C., Liu J., Liu C., He Y. (2018). Genome-wide investigation of *WRKY* gene family in pineapple: Evolution and expression profiles during development and stress. BMC Genom..

[12] Sahebi M., Hanafi M.M., Rafii M.Y., Mahmud T., Azizi P., Osman M., Abiri R., Taheri S., Kalhori N., Shabanimofrad M. (2018). Improvement of drought tolerance in rice (*Oryza sativa* L.): Genetics, genomic tools, and the *WRKY* gene family. BioMed Res. Int..

[13] Ding W., Ouyang Q., Li Y., Shi T., Li L., Yang X., Ji K., Wang L., Yue Y. (2020). Genome-wide investigation of WRKY transcription factors in sweet osmanthus and their potential regulation of aroma synthesis. Tree Physiol..

[14] Schluttenhofer C., Yuan L. (2015). Regulation of specialized metabolism by WRKY transcription factors. Plant Physiol..

[15] Yue H., Wang M., Liu S., Du X., Song W., Nie X. (2016). Transcriptome-wide identification and expression profiles of the WRKY transcription factor family in Broomcorn millet (*Panicum miliaceum* L.). BMC Genom..

[16] Eulgem T., Rushton P.J., Robatzek S., Somssich I.E. (2000). The WRKY superfamily of plant transcription factors. Trends Plant Sci..

[17] Rushton P.J., Somssich I.E., Ringler P., Shen Q.J. (2010). WRKY transcription factors. Trends Plant Sci..

[18] Mohanta T.K., Park Y.H., Bae H. (2016). Novel genomic and evolutionary insight of WRKY transcription factors in plant lineage. Sci. Rep..

[19] Chi Y., Yang Y., Zhou Y., Zhou J., Fan B., Yu J.Q., Chen Z. (2013). Protein-protein interactions in the regulation of WRKY transcription factors. Mol. Plant.

[20] Cackett L., Luginbuehl L.H., Schreier T.B., Lopez-Juez E., Hibberd J.M. (2022). Chloroplast development in green plant tissues: The interplay between light, hormone, and transcriptional regulation. New Phytol..

[21] Pandey S.P., Somssich I.E. (2009). The role of WRKY transcription factors in plant immunity. Plant Physiol..

[22] Ichimaru K., Yamaguchi K., Harada K., Nishio Y., Hori M., Ishikawa K. (2022). Cooperative regulation of PBI1 and MAPKs controls WRKY45 transcription factor in rice immunity. Nat. Commun..

[23] Wang C.T., Ru J.N., Liu Y.W., Li M., Zhao D., Yang J.F., Fu J.-D., Xu Z.S. (2018). Maize WRKY transcription factor ZmWRKY106 confers drought and heat tolerance in transgenic plants. Int. J. Mol. Sci..

[24] Cui Q., Yan X., Gao X., Zhang D.M., He H.B., Jia G.X. (2018). Analysis of WRKY transcription factors and characterization of two *Botrytis cinerea*-responsive *LrWRKY* genes from *Lilium regale*. Plant Physiol. Biochem. PPB.

[25] Zhao M.M., Zhang X.W., Liu Y.W., Li K., Tan Q., Zhou S., Wang G., Zhou C.J. (2020). A WRKY transcription factor, TaWRKY42-B, facilitates initiation of leaf senescence by promoting jasmonic acid biosynthesis. BMC Plant Biol..

[26] Zhou Y., Cheng Y., Wan C., Li J., Yang Y., Chen J. (2020). (Genome-wide characterization and expression analysis of the *Dof* gene family related to abiotic stress in watermelon. PeerJ.

[27] Wu W., Zhu S., Xu L., Zhu L., Wang D., Liu Y. (2022). Genome-wide identification of the Liriodendron chinense *WRKY* gene family and its diverse roles in response to multiple abiotic stress. BMC Plant Biol..

[28] Chen C., Chen H., Zhang Y., Thomas H.R., Frank M.H., He Y., Xia R. (2020). TBtools: An integrative toolkit developed for interactive analyses of big biological data. Mol. Plant.

[29] Hall B.G. (2013). Building phylogenetic trees from molecular data with MEGA. Mol. Biol. Evol..

[30] Waqas M., Azhar M.T., Rana I.A., Azeem F., Ali M.A., Nawaz M.A., Chung G., Atif R.M. (2019). Genome-wide identification and expression analyses of WRKY transcription factor family members from chickpea (*Cicer arietinum* L.) reveal their role in abiotic stress-responses. Genes Genom..

[31] Qu R., Cao Y., Tang X., Sun L., Wei L., Wang K. (2021). Identification and expression analysis of the *WRKY* gene family in *Isatis indigotica*. Gene.

[32] You J., Wang Y., Zhang Y., Dossa K., Li D., Zhou R. (2018). Genome-wide identification and expression analyses of genes involved in raffinose accumulation in sesame. Sci. Rep..

[33] Zhong Y., Wang P., Zhang X., Cheng Z.M. (2021). (2021). Recent duplications dominate VQ and WRKY gene expansions in six Prunus species. Int. J. Genom..

[34] Costa J.H., Bazioli J.M., de Moraes Pontes J.G., Fill T.P. (2019). *P. digitatum* infection mechanisms in citrus: What do we know so far?. Fungal Biol..

[35] Li L., Xin Z., Okwong R.O., OuYang Q., Che J., Zhou J., Tao N. (2021). Antofine inhibits postharvest green mold due to imazalil-resistant *P. digitatum* strain Pdw03 by triggering oxidative burst. J. Food Biochem..

[36] Wei Z., Zhang Z., Zhao W., Yin T., Liu X., Zhang H. (2022). Overexpression of *MET4* leads to the upregulation of stress-related genes and enhanced sulfite tolerance in *Saccharomyces uvarum*. Cells.

[37] Li Y.P., Zhang Z.M., Liu X.Z., Wei Z., Zhang X., Bian W., Li S., Zhang H. (2022). Transcriptome analysis of low-temperature-treated tetraploid yellow *Actinidia chinensis* Planch. Tissue culture plantlets. Life.

[38] Zhang J.S., Wang H.Q., Xia J., Sha K., He S.T., Dai H., Hao X.H., Zhou Y.W., Wang Q., Ding K.K. (2022). Coevolutionary insights between promoters and transcription factors in the plant and animal kingdoms. Zool. Res..

[39] Jores T., Tonnies J., Wrightsman T., Buckler E.S., Cuperus J.T., Fields S., Queitsch C. (2021). Synthetic promoter designs enabled by a comprehensive analysis of plant core promoters. Nat. Plants.

[40] He H., Dong Q., Shao Y., Jiang H., Zhu S., Cheng B., Xiang Y. (2012). Genome-wide survey and characterization of the *WRKY* gene family in *Populus trichocarpa*. Plant Cell Rep..

[41] Chen L., Song Y., Li S., Zhang L., Zou C., Yu D. (2012). The role of WRKY transcription factors in plant abiotic stresses. Biochim. Biophys. Acta.

[42] Chanwala J., Satpati S., Dixit A., Parida A., Giri M.K., Dey N. (2020). Genome-wide identification and expression analysis of WRKY transcription factors in pearl millet (*Pennisetum glaucum*) under dehydration and salinity stress. BMC Genom..

[43] Ashburner M., Ball C.A., Blake J.A., Botstein D., Butler H., Cherry J.M., Davis A.P., Dolinski K., Dwight S.S., Eppig J.T. (2000). Gene ontology: Tool for the unification of biology. The gene ontology consortium. Nat. Genet..

[44] Park C.J., Ronald P.C. (2012). Cleavage and nuclear localization of the rice XA21 immune receptor. Nat. Commun..

[45] Jia Y., Wang Z., Fjellstrom R.G., Moldenhauer K.A., Azam M.A., Correll J., Lee F.N., Xia Y., Rutger J.N. (2004). Rice Pi-ta gene confers resistance to the major pathotypes of the rice blast fungus in the United States. Phytopathology.

[46] Lippok B., Birkenbihl R.P., Rivory G., Brümmer J., Schmelzer E., Logemann E., Somssich I.E. (2007). Expression of *AtWRKY33* encoding a pathogen- or PAMP-responsive WRKY transcription factor is regulated by a composite DNA motif containing W box elements. Mol. Plant-Microbe Interact. MPMI.

[47] Lai Z., Vinod K., Zheng Z., Fan B., Chen Z. (2008). Roles of Arabidopsis WRKY3 and WRKY4 transcription factors in plant responses to pathogens. BMC Plant Biol..

[48] Hsu F.C., Chou M.Y., Chou S.J., Li Y.R., Peng H.P., Shih M.C. (2013). Submergence confers immunity mediated by the WRKY22 transcription factor in *Arabidopsis*. Plant Cell.

[49] Xing D.H., Lai Z.B., Zheng Z.Y., Vinod K.M., Fan B.F., Chen Z.X. (2008). Stress- and pathogen-induced *Arabidopsis* WRKY48 is a transcriptional activator that represses plant basal defense. Mol. Plant.

[50] Kim K.C., Fan B., Chen Z. (2006). Pathogen-induced Arabidopsis WRKY7 is a transcriptional repressor and enhances plant susceptibility to *Pseudomonas syringae*. Plant Physiol..

[51] Ulker B., Shahid Mukhtar M., Somssich I.E. (2007). The WRKY70 transcription factor of *Arabidopsis* influences both the plant senescence and defense signaling pathways. Planta.

[52] Fei X., Hou L., Shi J., Yang T., Liu Y., Wei A. (2018). Patterns of drought response of 38 WRKY transcription factors of *Zanthoxylum bungeanum* Maxim. Int. J. Mol. Sci..

[53] Yu Z., Zhang D., Zeng B., Liu X., Yang J., Gao W., Ma X. (2022). Characterization of the *WRKY* gene family reveals its contribution to the adaptability of almond (*Prunus dulcis*). PeerJ.

[54] Yue H., Chang X., Zhi Y., Wang L., Xing G., Song W., Nie X. (2019). Evolution and identification of the *WRKY* gene family in Quinoa (*Chenopodium quinoa*). Genes.

[55] Xu H., Watanabe K.A., Zhang L., Shen Q.J. (2016). WRKY transcription factor genes in wild rice *Oryza nivara*. DNA Res. Int. J. Rapid Publ. Rep. Genes Genomes.

[56] Ayadi M., Hanana M., Kharrat N., Merchaoui H., Marzoug R.B., Lauvergeat V., Rebaï A., Mzid R. (2016). The WRKY transcription factor family in *Citrus*: Valuable and useful candidate genes for Citrus breeding. Appl. Biochem. Biotechnol..

[57] Yamamoto S., Nakano T., Suzuki K., Shinshi H. (2004). Elicitor-induced activation of transcription via W box-related cis-acting elements from a basic chitinase gene by WRKY transcription factors in tobacco. Biochim. Biophys. Acta.

[58] Wei K.F., Chen J., Chen Y.F., Wu L.J., Xie D.X. (2012). Molecular phylogenetic and expression analysis of the complete WRKY transcription factor family in maize. DNA Res. Int. J. Rapid Publ. Rep. Genes Genomes.

[59] Villano C., Esposito S., D’Amelia V., Garramone R., Alioto D., Zoina A., Aversano R., Carputo D. (2020). *WRKY* genes family study reveals tissue-specific and stress-responsive TFs in wild potato species. Sci. Rep..

[60] Mukhtar M.S., Liu X., Somssich I.E. (2017). Elucidating the role of WRKY27 in male sterility in *Arabidopsis*. Plant Signal. Behav..

[61] Ali M.A., Azeem F., Nawaz M.A., Acet T., Abbas A., Imran Q.M., Shah K.H., Rehman H.M., Chung G., Yang S.H. (2018). Transcription factors WRKY11 and WRKY17 are involved in abiotic stress responses in *Arabidopsis*. J. Plant Physiol..

[62] Cheng X., Zhao Y., Jiang Q., Yang J., Zhao W., Taylor I.A., Peng Y.L., Wang D., Liu J. (2019). Structural basis of dimerization and dual W-box DNA recognition by rice WRKY domain. Nucleic Acids Res..

[63] Marè C., Mazzucotelli E., Crosatti C., Francia E., Stanca A.M., Cattivelli L. (2004). Hv-WRKY38: A new transcription factor involved in cold- and drought-response in barley. Plant Mol. Biol..

[64] Guo L., Li C., Jiang Y., Luo K., Xu C. (2020). Heterologous expression of poplar WRKY18/35 paralogs in *Arabidopsis* reveals their antagonistic regulation on pathogen resistance and abiotic stress tolerance via variable hormonal pathways. Int. J. Mol. Sci..

[65] Wang Z., Zhou Z., Liu Y., Liu T., Li Q., Ji Y. (2015). Functional evolution of phosphatidylethanolamine binding proteins in soybean and Arabidopsis. Plant Cell.

[66] Gu L., Li L., Wei H., Wang H., Su J., Guo Y., Yu S. (2018). Identification of the group IIa WRKY subfamily and the functional analysis of *GhWRKY17* in upland cotton (*Gossypium hirsutum* L.). PLoS ONE.

[67] Robatzek S., Somssich I.E. (2001). A new member of the *Arabidopsis* WRKY transcription factor family, AtWRKY6, is associated with both senescence- and defence-related processes. Plant J. Cell Mol. Biol..

[68] Wendel J.F. (2000). Genome evolution in polyploids. Plant Mol. Biol..

